# Phase I study of amrubicin plus cisplatin and concurrent accelerated hyperfractionated thoracic radiotherapy for limited‐disease small cell lung cancer: protocol of ACIST study

**DOI:** 10.1111/1759-7714.14555

**Published:** 2022-07-08

**Authors:** Kazumasa Akagi, Hirokazu Taniguchi, Minoru Fukuda, Takuya Yamazaki, Sawana Ono, Hiromi Tomono, Takayuki Suyama, Midori Shimada, Hiroshi Gyotoku, Shinnosuke Takemoto, Hiroyuki Yamaguchi, Yosuke Dotsu, Hiroaki Senju, Hiroshi Soda, Takashi Mizowaki, Yoshio Monzen, Takaya Ikeda, Seiji Nagashima, Yutaro Tasaki, Daisuke Nakamura, Kazutoshi Komiya, Katsumi Nakatomi, Eisuke Sasaki, Koichi Hirakawa, Hiroshi Mukae

**Affiliations:** ^1^ Department of Respiratory Medicine Nagasaki University Graduate School of Biomedical Sciences Nagasaki Japan; ^2^ Department of Respiratory Medicine Nagasaki Prefecture Shimabara Hospital Shimabara Japan; ^3^ Department of Radiology Nagasaki University Hospital Nagasaki Japan; ^4^ Clinical Research Center Nagasaki University Hospital Nagasaki Japan; ^5^ Clinical Oncology Center Nagasaki University Hospital Nagasaki Japan; ^6^ Department of Respiratory Medicine Sasebo City General Hospital Sasebo Japan; ^7^ Department of Radiology Sasebo City General Hospital Sasebo Japan; ^8^ Department of Respiratory Medicine National Hospital Organization Nagasaki Medical Center Omura Japan; ^9^ Department of Radiology National Hospital Organization Nagasaki Medical Center Omura Japan; ^10^ Department of Respiratory Medicine National Hospital Organization Ureshino Medical Center Ureshino Japan; ^11^ Department of Radiology National Hospital Organization Ureshino Medical Center Ureshino Japan

**Keywords:** amrubicin, chemotherapy, radiotherapy, small cell lung cancer

## Abstract

**Background:**

Etoposide plus cisplatin (EP) combined with concurrent accelerated hyperfractionated thoracic radiotherapy (AHTRT) is the standard treatment strategy for unresectable limited‐disease (LD) small cell lung cancer (SCLC), which has remained unchanged for over two decades. Based on a previous study that confirmed the non‐inferiority of amrubicin (AMR) plus cisplatin (AP) when compared with EP for extensive‐disease (ED) SCLC, we have previously conducted a phase I study assessing AP with concurrent TRT (2 Gy/time, once daily, 50 Gy in total) for LD‐SCLC therapy. Our findings revealed that AP with concurrent TRT could prolong overall survival to 39.5 months with manageable toxicities. Therefore, we plan to conduct a phase I study to investigate and determine the effect of AP combined with AHTRT, recommended dose (RD), maximum tolerated dose (MTD), and dose‐limiting toxicity (DLT) of AP in patients with LD‐SCLC.

**Methods:**

Treatment‐naive patients with LD‐SCLC, age between 20 and 75 years, who had a performance status of 0 or 1 and adequate organ functions will be enrolled. For chemotherapy, cisplatin 60 mg/m^2^/day (day 1) and AMR (day 1 to 3) will be administered with AHTRT (1.5 Gy/time, twice daily, 45 Gy in total). The initial AMR dose is set to 25 mg/m^2^/day. RD and MTD will be determined by evaluating toxicities.

**Discussion:**

Based on our previous study, the initial dose of AMR 25 mg/m^2^ is expected to be tolerated and acceptable. Here, we aim to determine whether treatment with AP and concurrent AHTRT would be an optimal choice with manageable toxicities for LD‐SCLC.

## Graphical abstract

Schema of ACIST study; phase I study to evaluate the efficacy and toxicity of CDDP + AMR + AHTRT for the patients with LD‐SCLC.

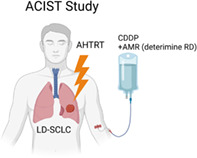

## INTRODUCTION

Small cell lung cancer (SCLC) accounts for ~13% of all lung cancers and is characterized by rapid growth, early metastasis, and high rates of relapse.^1^ Approximately one‐third of patients with SCLC are diagnosed with limited‐disease (LD)‐SCLC, and the treatment for patients with LD‐SCLC involves concurrent chemotherapy and radiotherapy, as <5% of these patients are suitable to undergo radical surgery.^2^, ^3^ Etoposide plus cisplatin (EP) combined with concurrent accelerated hyperfractionated thoracic radiotherapy (AHTRT) is considered the standard treatment for unresectable LD‐SCLC^4^, ^5^, ^6^; however, a high proportion of patients with LD‐SCLC experience disease relapse, accompanied by an insufficient long‐term survival rate. Accordingly, novel strategies are needed to achieve long‐term response and cure in patients with LD‐SCLC.

Amrubicin (AMR), or its active metabolite amurubicinol, exerts an antitumor effect by stabilizing the cleavable complex via topoisomerase II, which results in DNA cleavage and suppresses tumor growth.^7^ In treatment‐naive patients with extensive‐disease (ED)‐SCLC, AMR monotherapy has shown a remarkable improvement in overall response rate (ORR) (75.8%) and median overall survival (OS) (11.7 months).^8^ In addition, a clinical study has revealed the non‐inferiority of AMR plus cisplatin (AP) when compared with EP for treatment‐naive patients with ED‐SCLC in a Chinese population.^9^ These findings suggest the promising antitumor effect of AMR in treatment‐naive ED‐SCLC, which could also be used in LD‐SCLC. We previously conducted a phase I study to assess AP with concurrent TRT (2 Gy/time, once daily, 50 Gy in total) in patients with LD‐SCLC.^10^ Although the obtained results were from a limited patient cohort in a phase I study, AP with concurrent TRT markedly improved OS to 39.5 months. Dose‐limiting toxicities (DLTs) included neutropenia and leukopenia; however, all toxicities were manageable. Although this study indicated the promising results of AP with once‐daily concurrent TRT, no patient could achieve complete response, suggesting that further study will be required to “cure” the patients with LD‐SCLC.

Twice‐daily accelerated hyperfractionated irradiation (ie, 1.5 Gy/time, 5 days a week, 45 Gy in total) could significantly improve OS when compared with once‐daily irradiation (ie, 1.8 Gy/time, once daily, 5 days a week, 45 Gy in total)^4^; hence, the twice‐daily irradiation schedule could be tolerated and potentially prolong survival when combined with AP. Based on the previous studies, we plan to conduct a phase I study of AP combined with AHTRT (1.5 Gy/time, twice daily, 45 Gy in total) and aim to investigate and determine the effect, recommended dose (RD), maximum tolerated dose (MTD), and DLT of AP in patients with LD‐SCLC.

## METHODS

### Study design

**FIGURE 1:**
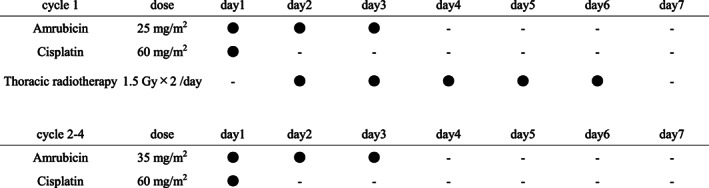
In the first cycle, AMR 25 (or 20, if reduced) mg/m^2^/day (from day 1 to day 3) and cisplatin 60 mg/m^2^/day (day 1) will be administered. In the following three cycles, AMR 35 mg/m^2^/day (from day 1 to day 3) and cisplatin 60 mg/m^2^/day (day 1) will be administered. The accelerated hyperfractionated thoracic radiotherapy, as 1.5 Gy/time, twice daily, 5 days a week, 45 Gy in total, will be administered concurrently from day 2 of the first chemotherapy cycle

This is a phase I, multi‐institutional, single‐arm, interventional study. The study design is shown in Figure 1. We initially plan to enroll three patients and evaluate the emergence of AMR‐related DLTs during the first cycle; the AMR dose is set at 25 mg/m^2^ for the first cycle of chemotherapy. DLTs will be confirmed if any of the following events occur: (i) grade 4 neutropenia or leukopenia for more than 4 days; (ii) grade 4 neutropenia or leukopenia with a fever of >38°C, suggesting an infection; (iii) grade 4 thrombocytopenia; and (iv) grade 3 or higher non‐hematologic toxicities. Adverse events will be assessed according to the National Cancer Institute Common Terminology Criteria for Adverse Events version 5.0.

**FIGURE 2:**
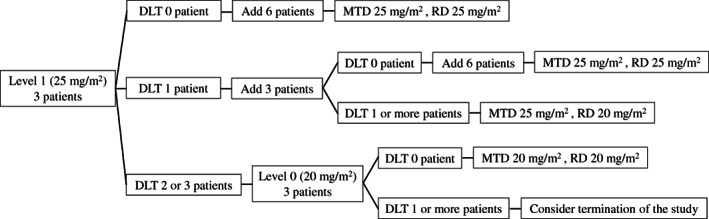
The schema of the method to determine RD and MTD in ACIST study. If DLTs are not observed in the initial three patients, we plan to add six patients. The 25 mg/m^2^ of AMR will be determined as MTD and RD. If DLTs are confirmed in one of the first three patients enrolled, we will add three additional patients at 25 mg/m^2^. If DLTs are confirmed in none of the additional three patients, we will add six additional patients. The 25 mg/m^2^ of AMR will be determined as MTD and RD. If DLTs are confirmed in more than one of the additional three patients, the 25 mg/m^2^ of AMR will be determined as MTD and the 20 mg/ml of AMR will be determined as RD. If DLTs are confirmed in more than two of the first three patients, we plan to add three patients to receive a reduced dose of 20 mg/m^2^. If DLTs are confirmed in none of the additional three patients, the 20 mg/m^2^ of AMR will be determined as MTD and RD. If DLTs are confirmed in more than one of the additional three patients, the study will be considered to terminate

MTD and RD will be determined as shown in Figure 2. (1) If DLTs are not observed in the initial three patients, we plan to add six patients as the extension study. The 25 mg/m^2^ of AMR will be determined as MTD and RD. (2) If DLTs are confirmed in one of the first three patients enrolled, we will add three additional patients at 25 mg/m^2^ of AMR to evaluate DLTs further. (2)‐1: If DLTs are confirmed in none of the additional three patients, we will add six additional patients as the extension study. The 25 mg/m^2^ of AMR will be determined as MTD and RD. (2)‐2: If DLTs are confirmed in more than one of the additional three patients, the study will be finished. The 25 mg/m^2^ of AMR will be determined as MTD and the 20 mg/m^2^ of AMR will be determined as RD. (3) If DLTs are confirmed in more than two of the first three patients, we plan to add three patients to receive a reduced dose of 20 mg/m^2^. (3)‐1: If DLTs are confirmed in none of the additional three patients, the 20 mg/m^2^ of AMR will be determined as MTD and RD. (3)‐2: If DLTs are confirmed in more than one of the additional three patients, the study will be considered to terminate.

All patients enrolled in the study will provide written informed consent, and the study will be conducted in accordance with the Declaration of Helsinki and Good Clinical Practice Guidelines. The study protocol was reviewed and approved by the clinical review board of Nagasaki University (CRB7180001). This study was registered in the Japan Registry of Clinical Trials (no. jRCT1071200033) on September 30, 2020.

### Inclusion criteria

(i) Histologically or cytologically confirmed SCLC (combined SCLC is acceptable); (ii) LD; (iii) age between 20 to 75 years; (iv) Eastern Cooperative Oncology Group Performance Status of 0 or 1 (v) regardless of measurable lesions; (vi) no prior chemotherapy; (vii) no multiple active cancers; (viii) latest clinical laboratory test within 14 days before registration meets the following criteria: (a) neutrophil count ≥1500 /μL, (b) platelet count ≥1.0 × 10^5^ /μL, (c) total‐bilirubin ≤1.5 mg/dL, (d) aspartate aminotransferase ≤100 U/L and alanine aminotransferase ≤100 U/L, (e) serum creatine ≤1.2 mg/dL, (f) SpO_2_ ≥ 92% (room air); (ix) radiologists expect sufficient preservation of respiratory function after chemoradiotherapy; and (x) written informed consent.

LD is defined as lesions limited to the ipsilateral thorax, contralateral mediastinum, and supraclavicular lymph nodes without malignant pleural or pericardial effusions.

### Exclusion criteria

(i) Resectable stage IA; (ii) presence of symptoms because of pericardial effusion; (iii) urgent radiation therapy is necessary because of superior vena cava syndrome; (iv) persistence of heart disease; (v) uncontrolled diabetes; (vi) uncontrolled hypertension; (vii) active systemic infection necessitating treatment; (viii) pregnant, possibly pregnant, postpartum within 28 days, breastfeeding, and males considering children; (ix) fever of 38.0°C; (x) mental disorder or symptoms interfering with daily life and inappropriate for enrollment; (xi) continuous intravenous or oral administration of steroids or immunosuppressants; (xii) hepatitis B surface antigen‐positive or hepatitis C virus antibody‐positive; (xiii) human immunodeficiency virus antibody‐positive (not necessarily examined); (xiv) interstitial pneumonia, pulmonary fibrosis, or emphysema confirmed by computed tomography; and (xv) other inappropriate complications.

### Interventions

#### Chemotherapy

In the first cycle, AMR 25 (or 20, if reduced) mg/m^2^/day (from day 1 to day 3) and cisplatin 60 mg/m^2^/day (day 1) will be administered. In the following three cycles, AMR 35 mg/m^2^/day (from day 1 to day 3) and cisplatin 60 mg/m^2^/day (day 1) will be administered (Figure 1).

#### Radiation therapy

AHTRT, as 1.5 Gy/time, twice daily, 5 days a week, 45 Gy in total, will be administered concurrently from day 2 of the first chemotherapy cycle (Figure 1). In addition to the three‐dimensional conformal radiation therapy, intensity‐modulated radiation therapy is acceptable. Calculating the actual value of V20 (volume of normal lung irradiated over 20 Gy compared to all lung volumes) or proportion of irradiation field for one lung is unnecessary before registration, and estimation by chest X‐ray or computed tomography is acceptable. To determine whether the desired respiratory function is preserved after radiotherapy, the target criterion of V20 is under 30%.

### Endpoints

The primary endpoint of the present study is to determine RD, and secondary endpoints are evaluating the response rate (RR), OS, progression‐free survival (PFS), MTD, DLTs and adverse effects. Efficacy will be assessed according to the Response Evaluation Criteria in Solid Tumors ver.1.1. PFS is defined as the time from the start date of chemotherapy to disease progression or death from any cause, whereas OS is defined as the time from the start date of chemotherapy to the last day confirmed to be alive or dead from any cause.

The Kaplan–Meier method will be used to estimate PFS and OS. Survival analysis will be conducted at the end of the follow‐up period. STATA SE version 16.1 (Stata Corp) will be used to perform analyses.

### Monitoring and follow‐up

Patients will be monitored twice yearly to ensure the accuracy and quality of the study. The principal investigator is responsible for compliance with the study protocol. The first three patients will be carefully monitored to determine whether the treatment was precisely conducted, data were collected, and serious nonconformities or adverse events occurred. Efficacy and toxicity will be followed up until the end of the follow‐up period.

## DISCUSSION

Several clinical trials have demonstrated the effects and toxicities of EP combined with AHTRT, exhibiting a median PFS between 15.4 and 18.8 months, median OS between 23.0 and 30.0 months, and manageable toxicities.^4^, ^5^, ^6^, ^11^ Although EP has been the standard regimen for patients with LD‐SCLC for two decades, novel therapeutic options are needed to further improve patient prognosis.

Regarding the combination of drugs other than EP with AHTRT, a phase III clinical study has compared EP and irinotecan plus cisplatin (IP) after induction of EP with AHTRT as consolidation therapy; however, three cycles of IP failed to improve OS when compared with three cycles of EP after one cycle of EP with concurrent AHTRT.^12^

In another study examining the consolidation of three cycles of AP after one cycle of EP with AHTRT, the authors documented an impressive 5‐year OS rate of 57.8%, suggesting the therapeutic potential of AP combined with AHTRT. The incidence of grade 3/4 neutropenia was 100%, whereas that of febrile neutropenia was 43%; however, all observed adverse effects were manageable with granulocyte‐colony stimulating factor support.^13^ It should be noted that the dose of AMR was set at 40 mg/m^2^ according to the RD from the phase I‐II study of AP for treatment‐naive ED‐SCLC,^14^ however, dose reduction was required in 33% of patients, implying that dose of 40 mg/m^2^ AMR might be too toxic for the patients with LD‐SCLC who will receive AP combined with AHTRT. Considering the toxicities associated with AMR, the RD of AMR with once‐daily TRT was evaluated from 20 mg/m^2^ in a dose‐escalation manner, and the RD of AMR was determined as 25 mg/m^2^ in our previous study.^7^ Following the introduction of twice‐daily irradiation, we plan to undertake a dose de‐escalation approach from 25 mg/m^2^ to determine the RD of AMR with twice‐daily AHTRT, based on our previous phase I study, which showed that doses of 25 mg/m^2^ AMR and 60 mg/m^2^ cisplatin are recommended for AP combined with TRT (2 Gy/day, once‐daily).^10^ We also set to raise the dose of AMR to 35 mg/m^2^ from second to fourth cycles of AP because AHTRT will finish during the first cycle of AP in this protocol. In a phase III study, which compared the efficacy of AP and that of IP in treatment naive Japanese patients with ED‐SCLC, the dose of AMR was reduced from 40 mg/m^2^ to 35 mg/m^2^ because of the high incidence of severe hematological toxicities.^15^ Furthermore, our retrospective study revealed that the real‐world incidence rate of febrile neutropenia among patients with thoracic malignancies that were treated with single‐agent AMR chemotherapy was 30%.^16^ Based on these findings for Japanese patients, the dose of 35 mg/m^2^ AMR from second cycle to fourth is predicted as the suitable dose in this protocol.

Sequential treatment with AMR or amurubicinol before ionizing radiation induces an additive radio‐enhancement effect in vitro.^17^ In addition, fractionation of a customary once‐daily dose into two treatments each day affords biological advantages, along with a high antitumor effect, resulting in the same total doses.^3^ The phase III CONVERT trial failed to demonstrate a survival advantage with high‐dose TRT when compared with standard twice‐daily TRT.^5^ Based on these findings, AP combined with twice‐daily AHTRT is expected to be a potential candidate for optimal treatment of LD‐SCLC.

Immunotherapy using immune checkpoint inhibitors (ICIs), such as programmed cell death 1 (PD‐1), programmed death‐ligand 1 (PD‐L1), and cytotoxic T‐lymphocyte‐associated protein 4, either as single agents or combined with cytotoxic chemotherapy, has revolutionized the treatment of several solid tumors, including SCLC.^18^ A recent study has shown that the addition of an anti‐PD‐1 antibody, pembrolizumab, to AMR for relapsed SCLC could afford a remarkable ORR of 52% and PFS rate of 14.4% at one year,^19^ indicating the promising effect of AMR when combined with ICIs. For some patients with LD‐SCLC who would benefit from ICIs, a combination with AP may be an optimal strategy in the future.

In summary, the present study is the first to evaluate AP combined with AHTRT for LD‐SCLC from the first chemotherapy cycle. This study will reveal whether AP combined with twice‐daily AHTRT could be tolerated and is an optimal regimen for LD‐SCLC.

## REGISTRATION DETAILS

This study was registered in the Japan Registry of Clinical Trials (no. jRCT1071200033) on September 30, 2020.

## AUTHOR CONTRIBUTIONS

All authors had full access to the protocol of the study and take responsibility for the integrity of the protocol. *Conceptualization*, M.F. *Investigation*, K.A., H.T. (Taniguchi), M.F., T.Y., H.T. (Tomono), S.O., T.S, M.S., H.G, S.T., H.Y., D.Y., H.S. (Senju), H.S. (Soda), T.M., Y.M., T.I., S.N., Y.T, D.N., K.K., K.N., E.S., K.H. *Methodology*, M. F. *Project Administration*, H.T. (Taniguchi) and M. F. *Supervision*, H. M. *Visualization*, K. A. *Writing‐ Original Draft Preparation*, K. A. and H.T. (Taniguchi). *Writing‐ Review and editing*, H.T. (Taniguchi), M.F., T.Y., H.T. (Tomono), S.O., T.S, M.S., H.G, S.T., H.Y., D.Y., H.S. (Senju), H.S. (Soda), T.M., Y.M., T.I., S.N., D.N., K.K., K.N., E.S., K.H., and H.M.

## DISCLOSURE

All the authors declare no potential conflicts of interest related to this work.
